# Profiles of Cognitive Functioning at 6 Months After Traumatic Brain Injury Among Patients in Level I Trauma Centers: A TRACK-TBI Study

**DOI:** 10.1001/jamanetworkopen.2023.49118

**Published:** 2023-12-26

**Authors:** Andrew M. Bryant, Nathan B. Rose, Nancy R. Temkin, Jason K. Barber, Geoffrey T. Manley, Michael A. McCrea, Lindsay D. Nelson, and the TRACK-TBI Investigators, Neeraj Badjatia, Shankar Gopinath, C. Dirk Keene, Christopher Madden, Laura B. Ngwenya, Ava Puccio, Claudia Robertson, David Schnyer, Sabrina R. Taylor, John K. Yue

**Affiliations:** 1 Department of Neurosurgery, Medical College of Wisconsin, Milwaukee; 2 Department of Neurology, The Ohio State University, Columbus; 3 Department of Neurological Surgery, University of Washington, Seattle; 4 Department of Biostatistics, University of Washington, Seattle; 5 Department of Neurological Surgery, University of California, San Francisco; 6 School of Medicine, University of Maryland, Baltimore; 7 Neurosurgery, Baylor College of Medicine, Houston, Texas; 8 Department of Laboratory Medicine and Pathology, University of Washington, Seattle; 9 Department of Neurological Surgery, UT Southwestern Medical Center, Dallas, Texas; 10 Department of Neurological Surgery, University of Cincinnati, Cincinnati, Ohio; 11 Department of Neurological Surgery, University of Pittsburgh, Pittsburgh, Pennsylvania; 12 Department of Neurosurgery, Baylor College of Medicine, Houston, Texas; 13 Department of Psychology, The University of Texas at Austin; 14 Department of Neurological Surgery, University of California, San Francisco

## Abstract

**Importance:**

Cognitive dysfunction is common after traumatic brain injury (TBI), with a well-established dose-response relationship between TBI severity and likelihood or magnitude of persistent cognitive impairment. However, patterns of cognitive dysfunction in the long-term (eg, 6-month) recovery period are less well known.

**Objective:**

To characterize the prevalence of cognitive dysfunction within and across cognitive domains (processing speed, memory, and executive functioning) 6 months after injury in patients with TBI seen at level I trauma centers.

**Design, Setting, and Participants:**

This prospective longitudinal cohort study used data from Transforming Research and Clinical Knowledge in TBI (TRACK-TBI) and included patients aged 17 years or older presenting at 18 US level I trauma center emergency departments or inpatient units within 24 hours of head injury, control individuals with orthopedic injury recruited from the same centers, and uninjured friend and family controls. Participants were enrolled between March 2, 2014, and July 27, 2018. Data were analyzed from March 5, 2020, through October 3, 2023.

**Exposures:**

Traumatic brain injury (Glasgow Coma Scale score of 3-15) or orthopedic injury.

**Main Outcomes and Measures:**

Performance on standard neuropsychological tests, including premorbid cognitive ability (National Institutes of Health Toolbox Picture Vocabulary Test), verbal memory (Rey Auditory Verbal Learning Test), processing speed (Wechsler Adult Intelligence Scale [4th edition] Processing Speed Index), and executive functioning (Trail Making Test).

**Results:**

The sample included 1057 persons with TBI (mean [SD] age, 39.3 [16.4] years; 705 [67%] male) and 327 controls without TBI (mean [SD] age, 38.4 [15.1] years; 222 [68%] male). Most persons with TBI demonstrated performance within 1.5 SDs or better of the control group (49.3% [95% CI, 39.5%-59.2%] to 67.5% [95% CI, 63.7%-71.2%] showed no evidence of impairment). Similarly, 64.4% (95% CI, 54.5%-73.4%) to 78.8% (95% CI, 75.4%-81.9%) of participants demonstrated no evidence of cognitive decline (defined as performance within 1.5 SDs of estimated premorbid ability). For individuals with evidence of either cognitive impairment or decline, diverse profiles of impairment across memory, speed, and executive functioning domains were observed (ie, the prevalence was >0 in each of the 7 combinations of impairment across these 3 cognitive domains for most TBI subgroups).

**Conclusions and Relevance:**

In this cohort study of patients seen at level I trauma centers 6 months after TBI, many patients with TBI demonstrated no cognitive impairment. Impairment was more prevalent in persons with more severe TBI and manifested in variable ways across individuals. The findings may guide future research and treatment recommendations.

## Key Points

**Question:**

What is the prevalence of distinct cognitive dysfunction profiles 6 months after traumatic brain injury (TBI) in patients at level I trauma centers?

**Findings:**

In this cohort study of 1057 patients with TBI, diverse cognitive impairment profiles were observed, with processing speed as the most commonly impaired domain in moderate-severe TBI and impairment prevalence lower and more equally distributed across domains in mild TBI. In this cohort, 64% to 79% performed commensurate with estimated premorbid abilities in memory, processing speed, and executive functioning.

**Meaning:**

Although many persons with TBI performed within normal limits cognitively 6 months after injury, those with cognitive dysfunction manifested diverse profiles, which may inform personalized interventions.

## Teaser

This cohort study examines the prevalence and characteristics of cognitive impairment and decline at 6 months after traumatic brain injury (TBI) among patients seen at level I trauma centers.

## Introduction

Traumatic brain injuries (TBIs) are common in the US and worldwide^1^ and are often associated with cognitive dysfunction^2^ in a dose-response manner relative to TBI severity. Patients with TBI often display reduced cognitive abilities^2,3,4^ followed by gradual recovery that is sometimes incomplete in the long-term (eg, 3- to 6-month) recovery period.^5,6,7,8^ Cognitive impairment after TBI is associated with quality of life even after accounting for TBI severity, premorbid or comorbid psychiatric symptoms, and sleep disturbance.^9^ Wilson et al^4^ found that cognitive impairment plays a key role in disability after TBI, particularly for individuals who are dependent on others for assistance and/or unable to participate in major life activities. Previous research has shown that impairments in processing speed, executive functioning, and memory are particularly common after TBI^2,7,10^; however, the frequency and specific patterns of cognitive dysfunction in this population are less well characterized.

Cognitive impairments following TBI are associated with functional independence,^11^ including likelihood of returning to work^12,13^ and driving.^14^ Cognition is not a unitary construct, and specific deficits can inform personalized interventions. For example, domain-specific cognitive abilities in the early phase of stroke are independent factors associated with long-term cognitive and functional outcomes.^15^ Patient engagement is an important factor associated with favorable outcomes of rehabilitation^16^; however, specific aspects of cognitive dysfunction (eg, low processing speed) may interfere with an individual’s ability to engage in such treatments. Guidelines suggest that cognitive rehabilitation should be tailored to an individual’s cognitive profile to prioritize restorative vs compensatory strategies.^17^ Despite these recommendations, previous literature has largely focused on characterizing differences in cognitive functioning between groups with vs without TBI to establish the presence or absence of cognitive dysfunction, with a focus on individual cognitive skills. Furthermore, prior work on cognitive outcomes of TBI^2^ has generally included small samples, focused on 1 severity group at a time (eg, mild TBI [mTBI]), or been retrospective. Uncertainty remains about the typical base rates of cognitive dysfunction across the TBI severity spectrum and especially the prevalence of different patterns of cognitive dysfunction across domains, which may be used in treatment decision-making and to target modifiable aspects of cognition (eg, stimulant medications for processing speed).^18^

This study leveraged data from a large, prospective sample of patients seen at level I trauma centers who had TBI at all severity levels to characterize long-term cognitive profiles. Data from the Transforming Research and Clinical Knowledge in TBI (TRACK-TBI) study were evaluated to address 2 aims: (1) to quantify the prevalence of impairment in different cognitive domains after mild to severe TBI at 6 months after injury and (2) to characterize the frequency of distinct profiles of cognitive impairment or decline across cognitive domains (processing speed, verbal memory, and executive functioning). By advancing understanding of the cognitive phenotypes after TBI, the findings may inform development of personalized interventions to mitigate the impact of cognitive dysfunction after TBI.

## Methods

### Participants and Study Design

This cohort study used data from TRACK-TBI, a large, prospective study recruiting participants at 18 US level I trauma centers. Participants aged 17 years or older with TBI across all severity levels who were enrolled between March 2, 2014, and July 27, 2018, were considered for inclusion in these analyses, which were conducted from March 5, 2020, through October 3, 2023. The study was approved by the institutional review board of each enrolling institution. All participants or their legally authorized representatives provided written informed consent. Demographic, injury, and outcome variables were collected in accordance with the TBI Common Data Elements.^19,20,21^ This study followed the Strengthening the Reporting of Observational Studies in Epidemiology (STROBE) reporting guideline.

For inclusion in this study, patients with TBI were required to have presented to a participating trauma center within 24 hours of injury with clinical indications for obtaining a computed tomography (CT) scan under American College of Emergency Medicine and US Centers for Disease Control and Prevention criteria. Data were obtained from control individuals with orthopedic injury and healthy friend and family controls enrolled between March 14, 2016, and August 2, 2019. Injury-related cognitive impairment was not expected at 6 months in the control group with orthopedic injury,^22^ which was combined with the healthy friend and family controls to achieve a single control group without TBI (normative comparison). Inclusion and exclusion criteria are described elsewhere.^23^ Race and ethnicity data were included in the analysis because of research conventions in the field of TBI; categories were Black or African American, White, other (Alaska Native or Inuit, American Indian or Native American, Asian, Native Hawaiian or Pacific Islander, and multiracial), and unknown. The source of race and ethnicity (eg, medical records, participant report) was not collected. All outcome measures were administered and scored by trained examiners certified on the TRACK-TBI outcome assessment battery, and participants were assessed with the measures of interest at 2 weeks, 6 months, and 12 months after injury. For the current study, participants with objective cognitive assessment data at 6 months and a premorbid estimate from at least 1 of the follow-up periods were included. A flow diagram is provided in the eFigure in Supplement 1.

### Measures

#### Premorbid Ability

Performance on the National Institutes of Health Toolbox Picture Vocabulary Test (hereafter, *vocabulary test*) was used to estimate premorbid cognitive ability. The vocabulary test is a measure of crystalized intelligence with high test-retest reliability (0.87) and validity (eg, robust correlations with other traditional neuropsychological measures used to estimate premorbid ability, such as the American National Adult Reading Test [*r* = 0.72]).^24^ Crystalized intelligence is generally resistant to injury or illness^25^; therefore, when the 6-month vocabulary test score was unavailable, data from other time points were used, in the following priority order: the mean of the 2-week and 12-month time points, the 12-month time point, or the 2-week time point.

#### Objective Cognitive Assessment

The TRACK-TBI cognitive assessment battery measured verbal episodic memory (Rey Auditory Verbal Learning Test [RAVLT] total score for trials 1-5 and delayed recall),^26^ processing speed (Wechsler Adult Intelligence Scale [4th Edition] Processing Speed Index [PSI]^27^ and Trail Making Test [TMT], part A), and executive functioning (TMT, part B and TMT B/A ratio).^28^ Higher scores on the measures of the RAVLT total score for trials 1 to 5 (possible range, 0-65) and delayed recall score (possible range, 0-15) and on the PSI (possible range, 50-150) indicate better performance. On the TMT, higher part A and B scores (ie, slower completion times) represent worse performance; higher B/A ratio scores reflect poorer executive functioning. Because administration allowed for discontinuation after 100 seconds (TMT-A) and 300 seconds (TMT-B), discontinued trials were coded as 101 and 301 seconds, respectively.

### Primary Outcome Measures

#### Cognitive Impairment and Cognitive Decline

We conceptualized 2 separate yet related adverse cognitive outcomes following TBI. Cognitive impairment was defined as performance substantially below demographically relevant normative levels using the control group without TBI to establish normative performance based on age, sex, and educational level. Cognitive decline was defined as performance substantially below estimated preinjury levels using vocabulary test scores to estimate premorbid general cognitive functioning. For this measure, the control group without TBI was also used to index performance on all tests compared with demographically appropriate normative values and to thereby put all cognitive measures onto the same scale (*z* score scale). Because cutoff scores for defining abnormal performance are somewhat arbitrary,^29^ we performed analyses at 3 common cutoffs: performance of 1.0 (16th percentile), 1.5 (7th percentile), and 2.0 (2nd percentile) SDs below expected levels.

#### Phenotypes or Profiles of Cognitive Dysfunction

In addition to characterizing the prevalence of adverse cognitive outcomes, we examined the prevalence of 8 distinct profiles of cognitive impairment or decline across the domains assessed. These included (1) no impairment or decline, (2) memory impairment or decline only (ie, impairment or decline only on 1 or both RAVLT indices), (3) processing speed impairment or decline only (ie, impairment or decline only on the PSI and/or TMT-A), (4) executive functioning impairment or decline only (ie, impairment or decline only on the TMT-B and/or TMT-B/A), (5) memory and processing speed impairment or decline (ie, met the aforementioned criteria for memory and processing speed impairment only), (6) memory and executive functioning impairment or decline, (7) processing speed and executive functioning impairment or decline, and (8) impairment or decline across all domains.

### Statistical Analysis

Individuals with TBI were classified into subgroups using common severity indicators: admission Glasgow Coma Scale (GCS) score and the presence (CT+) or absence (CT−) of acute intracranial findings on clinical head CT (ie, a GCS score of 13-15 and CT− was considered CT− mild TBI [mTBI], a GCS score of 13-15 and CT+ was considered CT+ mTBI, and a GCS score of 3-12 was considered moderate-severe TBI [msTBI]). Sample demographics and injury characteristics were summarized with descriptive statistics. Within the control group, linear regression models were used to estimate performance on each cognitive measure, including age, sex, and educational level. Age was coded as the number of years beyond 60; quadratic terms were nonsignificant and not included in the reported models. The resulting regression model for each measure was used to estimate demographically appropriate performance for individuals in the group with TBI and to then compute the difference between actual and normative performance in *z* score units. These *z* scores, reflecting an individual’s performance compared with normative performance, were dichotomized as impaired or not impaired using 3 cutoffs (*z* score<−1.0, *z* score<−1.5, and *z* score<−2.0). To establish cognitive decline, these *z*-scored performance levels were compared with participants’ estimated premorbid performance, operationalized as the normatively adjusted performance on the vocabulary test. Parallel to definitions of cognitive impairment, cognitive decline was classified as performance of 1.0, 1.5, and 2.0 SDs below participants’ own estimated premorbid ability levels (vocabulary normative *z* score). All primary analyses estimated percentages (95% CIs) using inverse probability weighting to account for potentially biased patterns of attrition. Weights were derived from a boosted regression algorithm modeling the propensity for having been assessed on the 6-month cognitive outcomes based on baseline demographic and injury characteristics. Analyses were performed using SPSS, version 27 (IBM Corp) except for propensity modeling, which was done using the Toolkit for Weighting and Analysis of Nonequivalent Groups boosted-regression software from the RAND Corporation.^30^

## Results

### Participants

The sample included 1057 participants with TBI (mean [SD] age, 39.3 [16.4] years; 352 [33%] female; 705 [67%] male). A total of 176 (17%) were Black or African American; 815 (77%), White; 64 (6%), other race and ethnicity; and 2 (0%), unknown race and ethnicity. Of 327 included controls without TBI (mean [SD] age, 38.4 [15.1] years; 105 [32%] female; 222 [68%] male), 50 (15%) were Black or African American; 250 (77%), White; 24 (7%), other race and ethnicity; and 3 (0%) unknown race and ethnicity. Demographic and injury characteristics for both groups are shown in Table 1.

**Table 1.** Demographics of Participants and Characteristics of Traumatic Brain Injury

Variable	Participants^a^					*P* value^d^
	Any TBI (n = 1057)	CT− mTBI (n = 634)^b^	CT+ mTBI (n = 316)^b^	msTBI (n = 107)^c^	Controls, no TBI (n = 327)	
Age, mean (SD) [range], y	39.3 (16.4) [17-83]	37.8 (15.6)	44.3 (17.6)	33.4 (13.8)	38.4 (15.1) [18-75]	<.001
Sex						
Female	352 (33)	242 (38)	87 (28)	23 (21)	105 (32)	<.001
Male	705 (67)	392 (62)	229 (72)	84 (79)	222 (68)	
Race^e^						
Black or African American	176 (17)	138 (22)	29 (9)	9 (8)	50 (15)	<.001
White	815 (77)	456 (72)	269 (85)	90 (84)	250 (77)	
Other^f^	64 (6)	38 (6)	18 (6)	8 (7)	24 (7)	
Unknown	2 (<1)	2 (0)	0	0	3 (<1)	
Ethnicity						
Hispanic	152 (14)	87 (14)	44 (14)	21 (20)	62 (19)	.09
Non-Hispanic	901 (86)	544 (86)	271 (86)	86 (80)	264 (81)	
Unknown	4 (<1)	3 (<1)	1 (<1)	0	1 (<1)	
Educational level, mean (SD), y^g^	13.9 (2.6)	13.9 (2.6)	14.2 (2.7)	13.2 (2.3)	14.3 (2.6)	<.001
Insurance type						
Government	202 (19)	125 (20)	64 (20)	13 (12)	62 (19)	.38
Private	812 (77)	483 (76)	237 (75)	92 (86)	249 (76)	
Other or unknown^h^	43 (4)	26 (4)	15 (5)	2 (2)	16 (5)	
Injury cause						
Assault	60 (6)	30 (5)	25 (8)	5 (5)	0	<.001
Fall	259 (25)	128 (20)	104 (33)	27 (25)	48 (15)	
MCC	91 (9)	47 (7)	27 (9)	17 (16)	19 (9)	
MVC						
Cyclist or pedestrian	200 (19)	115 (18)	70 (22)	15 (14)	13 (4)	
Vehicle occupant	316 (30)	239 (38)	46 (15)	31 (29)	19 (9)	
Other or unknown^i^	131 (12)	75 (12)	44 (14)	12 (11)	48 (15)	
None—friend and family controls	NA	NA	NA	NA	180 (55)	NA
GCS score at admission, mean (SD)	13.9 (2.7)	14.8 (0.5)	14.6 (0.6)	6.6 (3.4)	NA	NA
LOC, yes or suspected^j^	890 (89)	534 (87)	258 (88)	98 (98)	NA	.003
PTA, yes or suspected^k^	767 (80)	443 (76)	250 (85)	74 (95)	NA	<.001
Primary language						
English	1015 (96)	609 (97)	300 (95)	106 (99)	307 (94)	.12
Spanish	21 (2)	12 (2)	8 (3)	1 (1)	15 (5)	
Other or unknown^l^	17 (2)	10 (2)	7 (2)	0	5 (2)	

Abbreviations: CT−, negative findings on head computed tomography scan; CT+, positive findings on head CT scan; GCS, Glasgow Coma Scale; LOC, loss of consciousness; MCC, motorcycle crash; msTBI, moderate-severe traumatic brain injury; mTBI, mild TBI; MVC, motor vehicle crash; NA, not applicable; PTA, posttraumatic amnesia.

^a^ Data are presented as the number (percentage) of participants unless otherwise indicated. Because of rounding, percentages may not total to 100.

^b^ Admission Glasgow Coma Scale score of 13 to 15.

^c^ Admission Glasgow Coma Scale score of 3 to 12.

^d^ Statistical significance was assessed by the Kruskal-Wallis or Fisher exact test, as appropriate.

^e^ The source of race and ethnicity data (eg, medical records, participant report) was not collected.

^f^ Included Alaska Native or Inuit, American Indian or Native American, Asian, Native Hawaiian or Pacific Islander, and multiracial.

^g^ Unknown for 12 participants with TBI and 1 control participant (excluded from percentage calculations).

^h^ Any other type of health insurance or health coverage plan.

^i^ Other road traffic injury, other nonintentional injury, act of mass violence, suicide attempt.

^j^ Unknown for 52 participants with TBI (excluded from percentage calculations).

^k^ Unknown for 104 participants with TBI (excluded from percentage calculations).

^l^ Includes Amharic, Cantonese, French, Hindi, Italian, Mandarin, Persian, Romanian, Russian, Tagalog, other, and unlisted.

### Prevalence of Cognitive Impairment and Decline

**Figure 1.**
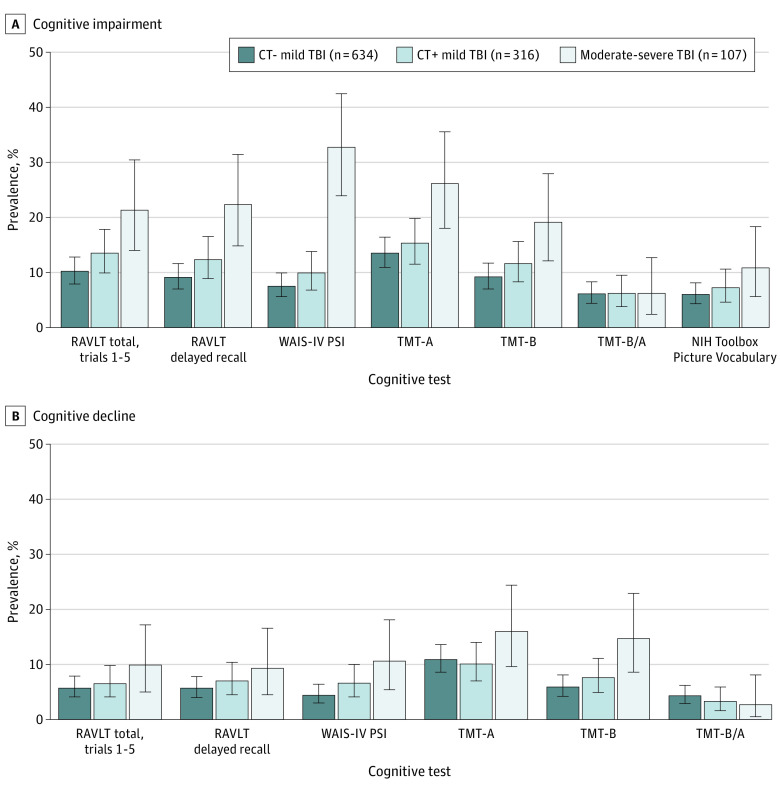
Prevalence of Cognitive Impairment and Cognitive Decline A, Performance more than 1.5 SDs below the normatively adjusted mean. B, Performance more than 1.5 SDs below estimated premorbid cognitive ability, based on the National Institutes of Health (NIH) Toolbox Picture Vocabulary Test. Whiskers represent 95% CIs. CT indicates computed tomography; RAVLT, Rey Auditory Verbal Learning Test; WAIS-IV PSI, Wechsler Adult Intelligence Scale (4th edition) Processing Speed Index; TMT-A, Trail Making Test, part A; TMT-B, Trail Making Test, part B; TMT-B/A, ratio of Trail Making Test B to A.

Descriptive statistics for primary cognitive outcome measures at 6 months are shown in Table 2. Figure 1 shows the percentage of participants with TBI with cognitive impairment and decline on individual measures at the 1.5-SD cutoff, stratified by TBI severity. Data at 3 cutoffs for cognitive dysfunction (1.0, 1.5, and 2.0 SDs below normative reference standards) are presented in the eTable 1 in Supplement 1. For simplicity, this summary focuses on the 1.5-SD cutoff (comparable to the 7th percentile). The percentage of participants with cognitive impairment (performance below normative reference standards) was generally higher for individuals with msTBI (6.2% [95% CI, 2.4%-12.7%] to 32.7% [95% CI, 23.9%-42.4%]) and lower for individuals with CT+ mTBI (6.2% [95% CI, 3.8%-9.5%] to 15.3% [95% CI, 11.5%-19.8%]) and CT− mTBI (6.1% [95% CI, 4.4%-8.3%] to 13.5% [95% CI, 10.9%-16.4%]). Within the group with msTBI, impairment rates were highest for measures of processing speed (26.1% [95% CI, 18.0%-35.5%] to 32.7% [95% CI, 23.9%-42.4%]) followed by memory (21.3% [95% CI, 14.0%-30.4%] to 22.3% [95% CI, 14.8%-31.4%]) and executive functioning (6.2% [95% CI, 2.4%-12.7%] to 19.1% [95% CI, 12.1%-27.9%]). Among participants with mTBI, there were minimal differences between the CT+ and CT− groups; the highest prevalence of impairment for both subgroups with mTBI was on TMT-A (13.5% [95% CI, 10.9%-16.4%] to 15.3% [95% CI, 11.5%-19.8%]).

**Table 2.** Descriptive Statistics for Primary Cognitive Outcome Measures at 6-Month Follow-Up

Variable	Score, Mean (SD)			
	CT− mild TBI^a^	CT+ mild TBI^a^	Moderate-severe TBI^b^	Controls, without TBI
NIH Toolbox Picture Vocabulary Test^c^	116 (12)	119 (12)	113 (10)	119 (12)
RAVLT total, trials 1-5	47 (11)	46 (11)	42 (11)	48 (11)
RAVLT, delayed recall	9.2 (3.4)	8.8 (3.5)	7.7 (3.8)	9.5 (3.5)
WAIS-IV PSI^d^	104 (15)	103 (16)	95 (17)	105 (15)
TMT				
Part A, s^e^	26 (11)	27 (14)	30 (14)	23 (9)
Part B, s^e^	68 (36)	72 (47)	81 (52)	63 (36)
B/A ratio^f^	2.73 (1.1)	2.67 (1.02)	2.78 (1.90)	2.81 (1.17)

Abbreviations: CT−, negative findings on head computed tomography scan; CT+, positive findings on head CT scan; RAVLT, Rey Auditory Verbal Learning Test; TBI, traumatic brain injury; TMT, Trail Making Test; WAIS-IV PSI, Wechsler Adult Intelligence Scale (4th edition) Processing Speed Index.

^a^ Admission Glasgow Coma Scale score of 13 to 15.

^b^ Admission Glasgow Coma Scale score of 3 to 12.

^c^ When 6-month data were unavailable or missing, the score represents the mean of the 2-week and 12-month, the 12-month, or the 2-week score (in the listed order of priority).

^d^ Mean (SD) value, 100 (15).

^e^ Longer completion times indicate poorer performance.

^f^ Higher ratio scores reflect poorer executive functioning.

Rates of cognitive decline (performance below one’s own estimated baseline cognitive functioning) showed a similar pattern across subgroups with TBI, with generally lower rates of cognitive decline than cognitive impairment (msTBI: 2.7% [95% CI, 0.5%-8.1%] to 16.0% [95% CI, 9.6%-24.4%] [mean, 10.5% (95% CI, 5.6%-17.9%)]; mTBI: 3.3% [95% CI, 1.6%-5.9%] to 10.9% [95% CI, 8.6%-13.6%] [mean, 6.5% (95% CI, 4.4%-9.3%)] for the 1.5-SD threshold for decline). Rates of both impairment and decline using TMT-B/A were low across all severity groups (2.7% [95% CI, 0.5%-8.1%] to 6.2% [95% CI, 2.4%-12.7%]), with no clear dose-response relationship with TBI severity.

### Prevalence of Cognitive Phenotypes

**Figure 2.**
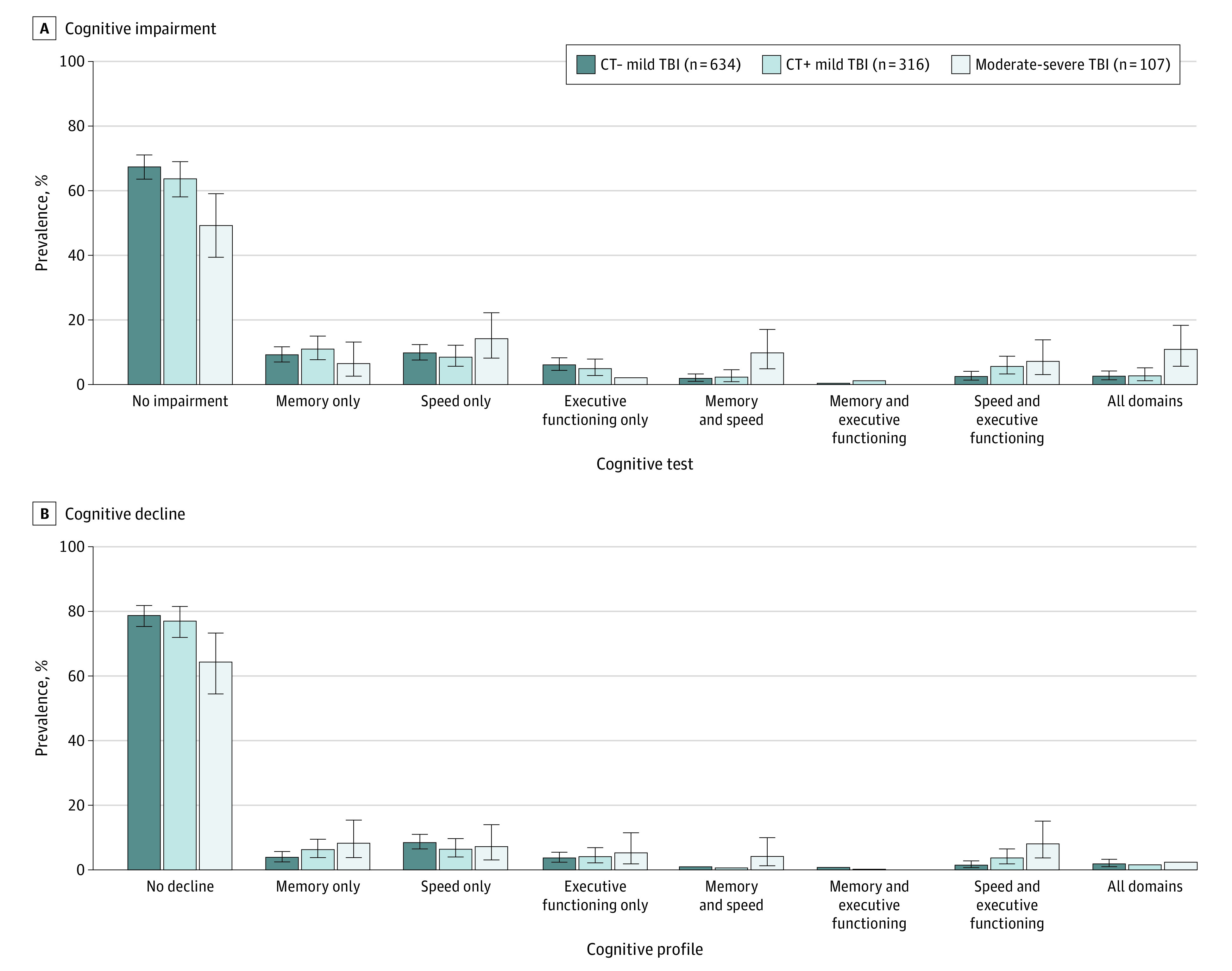
Prevalence of Different Profiles of Cognitive Impairment A, Performance more than 1.5 SDs below the normatively adjusted mean. B, Performance more than 1.5 SDs below estimated premorbid cognitive ability, based on a picture vocabulary test. Percentages may not total to 100 due to rounding. Whiskers represent 95% CIs.

eTable 2 in Supplement 1 provides the rates of specific profiles of cognitive dysfunction separately for each subgroup with TBI. These values are further illustrated in Figure 2 across TBI severity strata. Across the 3 cutoffs for cognitive dysfunction, the range of persons with no impairment was as follows: 49.1% (95% CI, 45.1%-53.0%) to 80.4% (95% CI, 77.0%-83.4%) for CT− mTBI, 44.7% (95% CI, 39.1%-50.3%) to 75.4% (95% CI, 70.2%-80.0%) for CT+ mTBI, and 41.7% (95% CI, 32.3%-51.7%) to 61.3% (95% CI, 51.4%-70.6%) for msTBI. When focusing on the middle (1.5 SD) cutoff for cognitive dysfunction, the prevalence of demonstrating no cognitive impairment across the 6 measures was higher for the group with mTBI (CT+: 63.8% (95% CI, 58.2%-69.1%) CT−: 67.5% (95% CI, 63.7%-71.2%) than the group with msTBI (49.3% [95% CI, 39.5%-59.2%]). Within the group with msTBI (1.5-SD cutoff), the prevalence rates of impaired profiles ranged from highest to lowest as follows: speed only (14.2% [95% CI, 8.2%-22.3%]), all domains (10.9% [95% CI, 5.7%-18.4%]), memory and speed (9.8% [95% CI, 4.9%-17.1%]), speed and executive functioning (7.2% [95% CI, 3.1%-13.9%]), memory only (6.5% [95% CI, 2.6%-13.2%]), and executive functioning only (2.1% [95% CI, 0.3%-7.0%]). In the subgroup with CT+ mTBI (1.5-SD cutoff), the top 3 impairment phenotypes were memory only (11.0% [95% CI, 7.7%-15.0%]), speed only (8.5% [95% CI, 5.7%-12.2%]), and speed and executive functioning (5.6% [95% CI, 3.3%-8.8%]), while the top 3 in the subgroup with CT− mTBI (1.5-SD cutoff) were speed only (9.8% [95% CI, 7.6%-12.4%]), memory only (9.2% [95% CI, 7.0%-11.7%]), and executive functioning only (6.1% [95% CI, 4.4%-8.3%]). The prevalence of global cognitive impairment (impairment in all 3 domains) was 2.6% (95% CI, 1.5%-4.2%) in the CT− mild TBI group, 2.7% (95% CI, 1.2%-5.2%) in the CT+ mild TBI group, and 10.9% (95% CI, 5.7%-18.4%) in the msTBI group. By cognitive domain, the prevalence of impairments in the msTBI group was as follows: processing speed (with or without other impairments), 42.1%; memory, 27.2%; and executive functioning, 20.2%. The prevalence in the mTBI groups by domain was as follows: processing speed, 16.8% to 19.1%; memory, 14.1% to 17.2%; and executive functioning, 11.6% to 14.4%.

For all subgroups with TBI, the most common profile of cognitive decline was no decline (CT− mTBI, 78.8% [95% CI, 75.4%-81.9%]; CT+ mTBI, 77.1% [95% CI, 72.0%-81.6%]; and msTBI, 64.4% [95% CI, 54.5%-73.4%], using the 1.5-SD cutoff). Although there was substantial heterogeneity in profiles of cognitive decline, at the 1.5-SD cutoff, decline in memory only was the most common profile of decline after msTBI (8.3% [95% CI, 3.8%-15.4%]), while decline in speed only was most common after mTBI (CT+: 6.4% [95% CI, 4.0%-9.7%]; CT−: 8.5% [95% CI, 6.5%-11.0%]). The prevalence of global cognitive decline was 1.9% (95% CI, 1.0%-3.3%) in the CT− mTBI group, 1.6% (95% CI, 1.0%-4.9%) in the CT+ mTBI group, and 2.4% (95% CI, 0.5%-7.4%) in the msTBI group.

## Discussion

This longitudinal cohort study of patients from 18 US level I trauma centers is, to our knowledge, the largest to report the prevalence of cognitive dysfunction at 6 months after injury across TBI severity levels. Cognitive dysfunction is a common consequence of TBI, yet 49.3% to 67.5% of participants exhibited no cognitive impairment (defined as >1.5 SDs below normative reference standards) across 6 objective measures of memory, processing speed, and executive functioning at 6 months after injury. Similarly, 64.4% to 78.8% of participants demonstrated no evidence of cognitive decline (ie, 1.5 SDs below estimated premorbid ability levels) on formal testing at this time point. The prevalence of adverse cognitive outcomes was largest after msTBI (GCS score of 3-12) and was more favorable and comparable among persons with CT− and CT+ mTBI (GCS score of 13-15). Overall, these findings are consistent with the established dose-response relationship between TBI severity and prevalence or degree of cognitive dysfunction in the long-term recovery phase (eg, 3-6 months).^31^ Although persons with msTBI had more unfavorable outcomes, the data showed that a minority of persons with msTBI had impairment across all individual measures of cognitive ability, many of which are commonly used in clinical neuropsychological evaluations.

Previous literature has largely focused on characterizing differences in cognitive functioning between a group with TBI and a control group and establishing the presence or absence of cognitive dysfunction on individual measures. Dichotomizing cognitive dysfunction as present vs absent dismisses the clinically relevant information in the overall cognitive profile of an individual. We observed a variety of distinct profiles of cognitive impairment in this sample. By 6 months after injury, rates of global cognitive impairment (ie, memory, processing speed, and executive functioning) were comparatively low (10.9% after msTBI and 2.6%-2.7% after mTBI, based on a 1.5-SD cutoff), indicating that patients with some level of cognitive impairment maintained strengths that may be leveraged to learn strategies to compensate for weaknesses.

Looking across domains of cognitive functioning, processing speed was the most impacted domain following msTBI (42.1% had impaired speed with or without other impairments vs 27.2% with impaired memory and 20.2% with impaired executive dysfunction). Impairment prevalence was more equally distributed in the groups with mTBI (16.8%-19.1%, 14.1%-17.2%, and 11.6%-14.4% of patients displayed speed, memory, and executive functioning impairment, respectively). Slow processing speed can persist for years after msTBI,^32^ has the strongest overall relationship with functional outcome,^4^ and has been identified as the most pronounced cognitive deficit observed 10 to 20 years following severe TBI.^31^ Overall, however, these findings showed substantial heterogeneity in cognitive functioning after TBI, which could have implications for clinical care and the design of treatment studies targeting cognition.^18^

Research suggests patients experience the greatest degree of cognitive improvement within the first year after injury, with relative cognitive stability from 1 to 10 years.^2,7,32,33,34^ We found that at 6 months after injury, a majority (64.4%-78.8%) of patients demonstrated cognitive functioning that was commensurate with their estimated premorbid abilities. This provides additional indirect support that the greatest recovery following injury likely occurs in the first several months, while providing valuable information on specific phenotypes of TBI-related cognitive sequelae that may be targeted with more comprehensive cognitive rehabilitation. Such knowledge may prove to be useful in reducing the long-term impact of cognitive sequelae following TBI (eg, decreasing functional impairments with adequate and appropriate rehabilitation).

A substantial proportion of patients with msTBI demonstrated no cognitive impairment or evidence of decline (49.3%-64.4% of individuals). On the other hand, 2.4% to 10.9% of individuals displayed global cognitive impairment or decline across the domains of memory, processing speed, and executive functioning, which may be particularly prognostic of poor outcome or poor response to treatment. Additional work is needed to link these profiles to other outcomes to better appreciate their significance.

Prior literature has suggested that positive acute intracranial findings on head CT are a poor prognostic indicator for outcomes following mTBI, especially return to normal function and early indices of neuropsychological functioning.^3,35,36^ In the sample in our study, patients with CT+ and CT− mTBI had broadly similar rates of cognitive dysfunction. The minimal differences in cognitive outcomes between participants with CT− and CT+ mTBI may be related to our focus on a time point (6 months) by which many individuals have returned to their preinjury cognitive baseline. Data from TRACK-TBI have previously shown that a substantial percentage of participants with CT− mTBI (27%) had acute neuroimaging findings on brain magnetic resonance imaging performed at about 2 weeks after injury.^21^ That is, the level I general population trauma center samples of participants with CT− mTBI likely reflect those with a generally higher severity of mTBI than found in other samples (eg, athletes). Additional research on the relationship between head CT and magnetic resonance imaging findings and specific cognitive impairments is warranted.

### Strengths and Limitations

Strengths of the study include the large, prospectively recruited sample, the inclusion of persons with TBI across all injury severity levels, the focus on clinically interpretable cognitive outcomes (impairment and decline) examined individually and in combination, and the use of controls without TBI to allow for evaluation of normative cognitive performance. Additionally, we presented prevalence data using a variety of thresholds for cognitive dysfunction, allowing these data to serve as a useful clinical reference.

Limitations of the study include a focus on patients seen at level I trauma centers who had undergone clinical head CT, which restricts generalizability. Additionally, we focused on 3 broad, historically defined subgroups with TBI, which did not allow for more nuanced understanding of the association between other clinically relevant injury data (eg, acute injury characteristics, neuroimaging features) and cognitive outcomes.

## Conclusions

The findings of this cohort study provide useful information on base rates of cognitive dysfunction and specific patterns of cognitive impairment in patients with TBI. Our data partially supported the established dose-response relationship^2^ between TBI severity and cognitive outcomes, with higher rates of cognitive dysfunction after msTBI (GCS score 3-12) than after mTBI (GCS score 13-15) but minimal differences between subgroups with CT+ and CT− mTBI. Importantly, we observed substantial heterogeneity in the profiles of cognitive dysfunction across the domains of verbal memory, processing speed, and executive functioning. The prevalence data provided can guide clinical judgments about patients’ cognitive performance and aid in the design of interventions to help persons with TBI compensate for and recover from long-term cognitive dysfunction. Future studies should investigate factors that predict distinct cognitive profiles and how cognitive rehabilitation or interventions can be tailored to patients’ cognitive strengths and weaknesses.

## Notes

Supplement 1. **eFigure.** CONSORT Diagram **eTable 1.** Percentage of Persons With TBI Who Met Different Thresholds for Cognitive Dysfunction **eTable 2.** Prevalence of Distinct Profiles of Cognitive Impairment or Decline at 6 Months After Injury, Stratified by TBI Severity

Supplement 2.
Data Sharing Statement
