# Supplementary material for: Profiles of Cognitive Functioning at 6 Months After Traumatic Brain Injury Among Patients in Level I Trauma Centers: A TRACK-TBI Study
Source: JAMA Netw Open. 2023 Dec 26;6(12):e2349118. doi: 10.1001/jamanetworkopen.2023.49118 (PMC10751593; doi:10.1001/jamanetworkopen.2023.49118)
Supplement: Supplement 1. — eFigure. CONSORT Diagram eTable 1. Percentage of Persons With TBI Who Met Different Thresholds for Cognitive Dysfunction eTable 2. Prevalence of Distinct Profiles of Cognitive Impairment or Decline at 6 Months After Injury, Stratified by TBI Severity [file jamanetwopen-e2349118-s001.pdf]

Supplementary Online Content

Bryant AM, Rose NB, Temkin NR, et al; TRACK-TBI Investigators. Profiles of cognitive functioning at 6 months after traumatic brain injury among patients in level 1 trauma centers: a TRACK-TBI study. *JAMA Netw Open*. 2023;6(12):e2349118. doi:10.1001/jamanetworkopen.2023.49118

eFigure. CONSORT Diagram
eTable 1. Percentage of Persons With TBI Who Met Different Thresholds for Cognitive Dysfunction
eTable 2. Prevalence of Distinct Profiles of Cognitive Impairment or Decline at 6 Months After Injury, Stratified by TBI Severity

This supplementary material has been provided by the authors to give readers additional information about their work.

© 2023 Bryant AM et al. *JAMA Network Open*.

eFigure. CONSORT diagram.

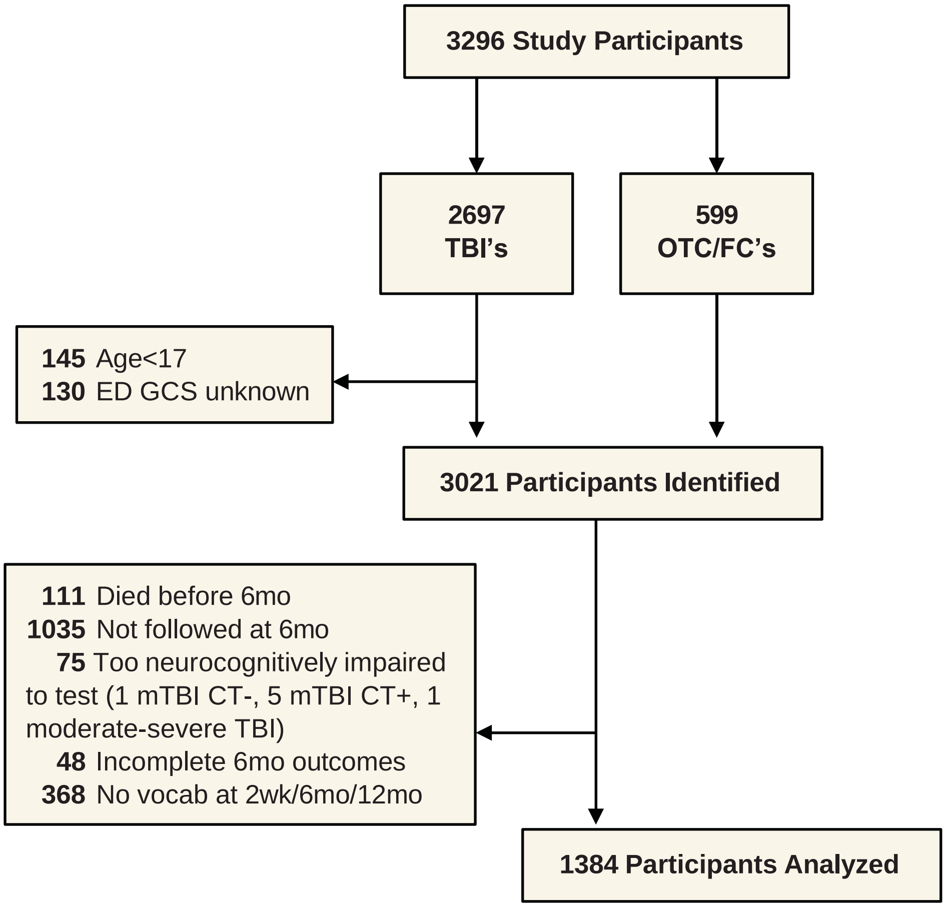

© 2023 Bryant AM et al. *JAMA Network Open*.

eTable 1. Percentage of Persons with TBI who met Different Thresholds (-1.0 Through -2.0 SD) for Cognitive Dysfunction

	**Mild TBI CT-**			**Mild TBI CT+**			**Moderate/Severe TBI**		
**Impairment,[1] % (95% CI)**	< 1 SD	< 1.5 SD	< 2 SD	< 1 SD	< 1.5 SD	< 2 SD	< 1 SD	< 1.5 SD	< 2 SD
NIH Toolbox Picture Vocabulary	17.3 (14.4, 20.4)	5.8 (4.1, 7.9)	1.7 (0.8, 3.1)	16.1 (12.2, 20.6)	7.0 (4.4, 10.4)	2.5 (1.1, 5.0)	20.9 (13.6, 29.8)	10.6 (5.4, 18.1)	4.0 (1.1, 9.8)
RAVLT Total 1-5	20.4 (17.3, 23.7)	10.2 (7.9, 12.8)	4.3 (2.9, 6.2)	25.3 (20.6, 30.5)	13.5 (9.9, 17.8)	7.6 (4.9, 11.1)	29.7 (21.2, 39.4)	21.3 (14.0, 30.4)	14.8 (8.6, 23.0)
RAVLT Delay	21.1 (18.0, 24.5)	9.1 (7.0, 11.6)	3.5 (2.2, 5.3)	23.5 (18.9, 28.6)	12.3 (8.9, 16.5)	8.5 (5.6, 12.1)	31.0 (22.4, 40.7)	22.3 (14.8, 31.4)	13.6 (7.7, 21.5)
WAIS-IV PSI	20.0 (17.0, 23.3)	7.5 (5.6, 9.9)	4.0 (2.6, 5.8)	22.5 (18.0, 27.5)	9.9 (6.8, 13.8)	4.9 (2.8, 7.9)	38.9 (29.6, 48.8)	32.7 (23.9, 42.4)	17.8 (11.1, 26.4)
TMT-A	20.4 (17.4, 23.8)	13.5 (10.9, 16.4)	9.3 (7.2, 11.9)	20.4 (16.1, 25.3)	15.3 (11.5, 19.8)	9.2 (6.2, 12.9)	35.7 (26.6, 45.5)	26.1 (18.0, 35.5)	17.9 (11.1, 26.5)
TMT-B	15.9 (13.1, 19.0)	9.2 (7.0, 11.7)	5.9 (4.2, 8.0)	13.4 (9.9, 17.7)	11.6 (8.3, 15.6)	9.0 (6.1, 12.8)	24.6 (16.8, 33.9)	19.1 (12.1, 27.9)	16.5 (10.0, 24.9)
TMT-B/A	10.2 (8.0, 12.9)	6.1 (4.4, 8.3)	4.4 (3.0, 6.3)	10.2 (7.1, 14.1)	6.2 (3.8, 9.5)	2.6 (1.1, 5.1)	10.9 (5.6, 18.4)	6.2 (2.4, 12.7)	4.0 (1.1, 9.7)
**Decline,[2] % (95% CI)**	< 1 SD	< 1.5 SD	< 2 SD	< 1 SD	< 1.5 SD	< 2 SD	< 1 SD	< 1.5 SD	< 2 SD
RAVLT Trial 1-5	12.9 (10.4, 15.8)	5.7 (4.1, 7.9)	2.0 (1.0, 3.4)	13.8 (10.1, 18.1)	6.5 (4.1, 9.8)	4.5 (2.5, 7.4)	18.8 (11.9, 27.6)	9.9 (5.0, 17.2)	2.3 (0.3, 7.5)
RAVLT Delayed Recall	11.1 (8.7, 13.8)	5.7 (4.0, 7.8)	2.4 (1.3, 3.9)	11.7 (8.3, 15.7)	7.0 (4.5, 10.4)	0.6 (0.0, 2.4)	17.1 (10.5, 25.7)	9.3 (4.5, 16.6)	1.8 (0.1, 6.8)
WAIS-IV PSI	10.4 (8.1, 13.1)	4.4 (3.0, 6.4)	1.2 (0.5, 2.4)	8.7 (5.9, 12.4)	6.6 (4.1, 10.0)	2.8 (1.2, 5.3)	26.2 (18.1, 35.6)	10.6 (5.4, 18.1)	4.8 (1.6, 10.8)
TMT-A	16.7 (13.9, 19.9)	10.9 (8.6, 13.6)	7.3 (5.4, 9.6)	13.9 (10.2, 18.2)	10.1 (7.0, 14.0)	6.9 (4.3, 10.3)	24.1 (16.3, 33.4)	16.0 (9.6, 24.4)	11.9 (6.4, 19.6)
TMT-B	11.0 (8.7, 13.7)	5.9 (4.2, 8.1)	4.2 (2.7, 6.0)	9.1 (6.2, 12.9)	7.6 (4.9, 11.1)	6.7 (4.2, 10.1)	15.9 (9.5, 24.3)	14.7 (8.6, 22.9)	11.5 (6.1, 19.2)
TMT-B/A	7.6 (5.7, 10.0)	4.3 (2.9, 6.2)	2.4 (1.4, 3.9)	5.2 (3.0, 8.3)	3.3 (1.6, 5.9)	1.5 (0.5, 3.6)	5.1 (1.8, 11.2)	2.7 (0.5, 8.1)	2.7 (0.5, 8.1)

Abbreviations. CT-, negative findings on head CT scan; CT+, positive findings on head CT scan; RAVLT, Rey Auditory Verbal Learning Test; RAVLT Total, trials 1-5; RAVLT Delay, delayed recall; TBI, traumatic brain injury; TMT, Trail Making Test; WAIS-IV PSI, Weschler Adult Intelligence Scale, Fourth Edition, Processing Speed Index.

[1]Cognitive impairment defined as normatively adjusted performance of z < - 1.0, -1.5, or -2.0.

[2]Cognitive decline defined as cognitive performance less than 1.0, 1.5, or 2.0 standard deviations below estimated premorbid ability levels (i.e., NIH Toolbox Picture Vocabulary test performance winsorized at a standard score of 115.

© 2023 Bryant AM et al. *JAMA Network Open*.

eTable 2. Prevalence of Distinct Profiles of Cognitive Impairment or Decline at 6 Months After Injury, Stratified by TBI Severity

Profile	Prevalence, % (95% CI)								
	CT− mild TBI[a]			CT+ mild TBI[a]			Moderate-severe TBI[b]		
	<1 SD	<1.5 SD	<2 SD	<1 SD	<1.5 SD	<2 SD	<1 SD	<1.5 SD	<2 SD
**Impairment phenotype**[c]									
No impairment	49.1 (45.1-53.0)	67.5 (63.7-71.2)	80.4 (77.0-83.4)	44.7 (39.1-50.3)	63.8 (58.2-69.1)	75.4 (70.2-80.0)	41.7 (32.3-51.7)	49.3 (39.5-59.2)	61.3 (51.4-70.6)
Memory only	12.9 (10.4-15.8)	9.2 (7.0-11.7)	3.7 (2.3-5.4)	17.2 (13.2-21.9)	11.0 (7.7-15.0)	8.4 (5.5-12.0)	10.7 (5.4-18.2)	6.5 (2.6-13.2)	8.1 (3.7-15.1)
Speed only	12.6 (10.1-15.5)	9.8 (7.6-12.4)	7.3 (5.4-9.6)	11.0 (7.8-15.0)	8.5 (5.7-12.2)	5.6 (3.3-8.8)	12.2 (6.7-20.0)	14.2 (8.2-22.3)	10.4 (5.3-17.8)
Executive functioning only	5.6 (4.0-7.7)	6.1 (4.4-8.3)	4.4 (2.9-6.3)	5.5 (3.2-8.6)	4.9 (2.8-7.9)	5.0 (2.9-8.1)	1.1 (0.0-5.4)	2.1 (0.3-7.0)	1.9 (0.2-6.7)
Memory and speed	5.4 (3.7-7.4)	1.9 (1.0-3.3)	0.5 (0.1-1.5)	8.1 (5.4-11.7)	2.3 (0.9-4.6)	0.5 (0.0-2.0)	9.7 (4.7-17.0)	9.8 (4.9-17.1)	3.7 (0.9-9.5)
Memory and executive functioning	2.7 (1.6-4.4)	0.4 (0.1-1.3)	0.3 (0.0-1.2)	2.2 (0.8-4.5)	1.2 (0.3-3.1)	0.4 (0.0-1.9)	0.0 (0.0-3.4)	0.0 (0.0-3.4)	1.1 (0.0-5.4)
Speed and executive functioning	5.1 (3.6-7.2)	2.5 (1.4-4.1)	2.0 (1.0-3.4)	5.7 (3.4-8.9)	5.6 (3.3-8.8)	3.0 (1.4-5.6)	7.9 (3.5-14.8)	7.2 (3.1-13.9)	6.1 (2.4-12.5)
All domains	6.5 (4.7-8.7)	2.6 (1.5-4.2)	1.5 (0.7-2.8)	5.6 (3.3-8.7)	2.7 (1.2-5.2)	1.8 (0.6-3.9)	16.8 (10.2-25.3)	10.9 (5.7-18.4)	7.4 (3.1-14.2)
**Decline phenotype**[d]									
No decline	63.9 (60.0-67.7)	78.8 (75.4-81.9)	85.9 (82.9-88.5)	68.8 (63.3-73.8)	77.1 (72.0-81.6)	84.9 (80.5-88.7)	49.9 (40.1-59.8)	64.4 (54.5-73.4)	78.6 (69.6-86.0)
Memory only	7.9 (5.9-10.3)	3.9 (2.5-5.7)	2.5 (1.4-4.0)	10.2 (7.1-14.1)	6.3 (3.8-9.5)	4.6 (2.6-7.5)	8.9 (4.2-16.0)	8.3 (3.8-15.4)	1.8 (0.1-6.8)
Speed only	10.9 (8.6-13.6)	8.5 (6.5-11.0)	6.1 (4.4-8.3)	6.5 (4.0-9.8)	6.4 (4.0-9.7)	2.8 (1.2-5.2)	15.9 (9.4-24.3)	7.2 (3.1-14.0)	4.8 (1.6-10.8)
Executive functioning only	5.4 (3.8-7.5)	3.7 (2.4-5.5)	3.2 (1.9-4.8)	3.4 (1.7-6.1)	4.1 (2.2-6.9)	3.0 (1.4-5.6)	2.0 (0.2-6.9)	5.3 (1.9-11.5)	6.8 (2.8-13.4)
Memory and speed	2.0 (1.1-3.5)	1.0 (0.4-2.1)	0.4 (0.1-1.4)	2.5 (1.1-5.0)	0.6 (0.0-2.3)	0.0 (0.0-1.2)	8.4 (3.8-15.5)	4.2 (1.3-10.0)	2.3 (0.3-7.5)
Memory and executive functioning	1.7 (0.9-3.1)	0.8 (0.2-1.8)	0.4 (0.1-1.3)	1.6 (0.5-3.7)	0.2 (0.0-1.7)	0.0 (0.0-1.2)	0.0 (0.0-3.4)	0.0 (0.0-3.4)	0.0 (0.0-3.4)
Speed and executive functioning	3.3 (2.0-5.0)	1.5 (0.7-2.8)	0.9 (0.3-2.0)	4.5 (2.5-7.4)	3.7 (1.9-6.5)	4.5 (2.5-7.4)	8.3 (3.8-15.4)	8.1 (3.7-15.1)	5.8 (2.1-12.1)
All domains	4.8 (3.3-6.8)	1.9 (1.0-3.3)	0.7 (0.2-1.7)	2.5 (1.0-4.9)	1.6 (0.5-3.7)	0.2 (0.0-1.7)	6.6 (2.7-13.1)	2.4 (0.4-7.4)	0.0 (0.0-3.4)

Abbreviations: CT, computed tomography; TBI, traumatic brain injury.

[a]Admission Glasgow Coma Scale score of 13 to 15.

[b]Admission Glasgow Coma Scale score of 3 to 12.

[c]Cognitive impairment was defined as a normatively adjusted performance of a z score less than –1.0, −1.5, or −2.0.

[d]Cognitive decline was defined as cognitive performance less than 1.0, 1.5, or 2.0 SDs below estimated premorbid ability levels (ie, National Institutes of Health Toolbox Picture Vocabulary Test performance winsorized at a standard score of 115).

© 2023 Bryant AM et al. *JAMA Network Open*.
