# Supplementary material for: Profiles of Cognitive Functioning at 6 Months After Traumatic Brain Injury Among Patients in Level I Trauma Centers: A TRACK-TBI Study
Source: JAMA Netw Open. 2023 Dec 26;6(12):e2349118. doi: 10.1001/jamanetworkopen.2023.49118 (PMC10751593; doi:10.1001/jamanetworkopen.2023.49118)
Supplement: Supplement 2. — Data Sharing Statement [file jamanetwopen-e2349118-s002.pdf]

Data Sharing Statement

Bryant. Profiles of Cognitive Functioning at 6 Months After Traumatic Brain Injury Among Patients in Level I Trauma Centers. *JAMA Netw Open*. Published December 26, 2023. doi:10.1001/jamanetworkopen.2023.49118

Data
Data available: Yes
Data types: Deidentified participant data
How to access data: The data are in FITBIR - the Federal Interagency TBI Research informatics system
When available: With publication

Supporting Documents
Document types: None

Additional Information
Who can access the data: Anyone who completes the FITBIR process for obtaining data.
Types of analyses: For research purposes per FITBIR policies.
Mechanisms of data availability: By mechanisms established by the FITBIR informatics system.
